# Global and local perspectives on tobacco harm reduction: what are the issues and where do we go from here?

**DOI:** 10.1186/s12954-018-0239-5

**Published:** 2018-06-22

**Authors:** Sharon Cox, Lynne Dawkins

**Affiliations:** Centre for Addictive Behaviours Research, School of Applied Sciences London South Bank University, 103 Borough Road, London, SE1 0AA UK

This thematic series of *Harm Reduction Journal* explores the issues surrounding the current state of tobacco harm reduction at global and local levels. Tobacco harm reduction (THR) refers to strategies designed to reduce the health risks associated with tobacco smoking but which may involve the continued use of nicotine. The health consequences of tobacco smoking are well documented; if current trends continue, it has been estimated that globally, a billion lives will be lost to tobacco smoking in the twenty-first century [1]. However, although some countries have embraced the concept, THR has not been, and is not, widely accepted or implemented, with heavy sanctions on reduced risk nicotine-containing products (including e-cigarettes, snus and heat not burn products) in many countries. Mike Russell famously stated ‘smokers smoke for the nicotine, but die from the tar’ [2]. Despite the ringing clarity of this message that it is the thousands of toxicants and numerous carcinogens in tobacco smoke that leads to premature death and disease, many years on, the science of ‘cleaner’ nicotine-containing products remains heavily contested. Nicotine’s long association with combustible tobacco continues to mar social political dialect surrounding the use of nicotine products for harm reduction.

Quitting smoking is a difficult process; high rates of relapse are testament to this. For example, 6–12-month abstinence rates with no intervention are only around 3%, with an estimated percentage point increase of between 6 and 15% with nicotine replacement therapy (NRT), buproprion or varenicline [3]. NRT may not adequately assist with all of the symptoms of smoking withdrawal, and for many more it is not an adequate long-term smoking replacement. E-cigarettes address the behavioural aspects of smoking in addition to providing nicotine and are now the most preferred method of quitting smoking [4]. However, when highlighting the potential of e-cigarettes for smoking cessation, in the absence of supporting empirical evidence, it is important not to overstate their achievements compared to NRT [5].

Although there is emerging evidence for their effectiveness as a smoking cessation aid, many smokers continue to smoke alongside e-cigarette use [6–9], or report that e-cigarettes are not a satisfying alternative to smoking [10]. Moreover, smoking prevalence remains disproportionately high in underrepresented (e.g. vulnerable/marginalised) groups (estimates of above 80% in those dependence on illicit substances) [11], and the effectiveness of e-cigarettes and other reduced risk nicotine-containing products have not been extensively researched in these groups. While so many smokers are still attached to smoking, more choice and availability of a range of both medicinal and consumer reduced risk nicotine-containing products is needed including NRT, e-cigarettes, snus, heat not burn products along with a greater understanding of how products may complement each other.

The development and maintenance of smoking is underpinned by a multifaceted and often complex set of interacting biopsychosocial issues, not captured by one theory. Experts in substance use know this. Despite this knowledge, one of the most commonly cited objections to reduced risk products is that because they contain nicotine they may act as a so-called gateway to regular cigarette smoking for non-smokers. However, one featured study shows that regular e-cigarette use is almost exclusive to ex and current smokers [9]. While this and other studies may help to counter claims of ‘gateway’ theories, this study along with past research shows that dual-use (i.e. use of an e-cigarette and smoking) is high. Why smokers with access to reduced risk products would still want to smoke and what factors encourage complete to switching to reduced harm products are key areas requiring empirical investigation.

Exploring quitting trajectories (which are presented in this issue) through qualitative research provides a more nuanced approach to understanding individual differences in smoking cessation. At a local level (which this thematic series invites), the narratives of those averse to change, those who have changed and those who have failed to switch, elucidate the challenges of behaviour change and also how nationwide strategies are perceived.

This edition features timely reminders of why the wider public perception of nicotine is so often skewed and marred by its association with tobacco [12]. Indeed, despite an emerging reduced risk market, public perception around the risks of these products is often inaccurate and the number of people incorrectly believing that e-cigarettes are as harmful or more harmful than smoking seems to be increasing [7]. This issue features one article which highlights how systems of communicating harms of smokeless tobacco originated and may continue to pollute the messaging of other nicotine-containing products. Tobacco health warning labels and risk-related messages have received a great deal of empirical attention in relation to their persuasive impact on driving down levels of smoking. With the landscape of nicotine use changing regulation has not kept up, messages on cigarettes appear to have been ‘borrowed’ and adapted, perhaps inappropriately, for use on much less harmful products. Despite these changes, the unintended consequences of warning labels on smokers’ perceptions of reduced risk products remain an emerging field.

Understanding the conditions under which smokers switch to e-cigarettes or other reduced risk products is clearly a priority area for research but one that may be exclusive to countries where regulation is relaxed, markets are developing and real world user behaviour can contribute to scientific evidence. The bigger picture is that there are still many countries with extremely high smoking prevalence rates with little or no access to reduced risk products. Where THR goes from here is unclear and is dependent on many factors. Perceptions and tolerance of nicotine vary widely between countries. Selective reporting, misrepresentation and/or misunderstanding of evidence further muddy the water. This may be compounded by a distrust of the tobacco industry and scepticism over its involvement and role in harm reduction. Nevertheless, opposition to harm reduction and tensions that exist between the scientific, industry and political communities on the issue of e-cigarettes and other reduced risk products ultimately means users lose out.

While the evidence on the reduced harm status of e-cigarettes, snus and other non-combustible nicotine-containing products continues to accumulate, there are those who continue to focus on the uncertainties. Tobacco harm reduction, although not risk free, is likely to reap considerable public health benefits but for its potential to be realised, a paradigm shift is required; there is no place for intolerance to nicotine per se when smoking-related death and disease continue unabated across the globe.
