# Correction to: Global and local perspectives on tobacco harm reduction: what are the issues and where do we go from here?

**DOI:** 10.1186/s12954-018-0242-x

**Published:** 2018-07-16

**Authors:** Sharon Cox, Lynne Dawkins

**Affiliations:** Centre for Addictive Behaviours Research, School of Applied Sciences London South Bank University, 103 Borough Road, London, SE1 0AA UK

## Correction

After publication of the original article [1], the authors noticed an error in the Competing interests section. The sentence “MG received a research grant from Pfizer and serves on an advisory board to Johnson & Johnson, manufacturers of smoking cessation medications” should not have been included in the section. As such, the correct statement should be as follows:

**Competing interests**

SC has no competing interests to declare. LD has conducted research for independent electronic cigarette companies. These companies had no input into the design, conduct or write up of the projects. She has also acted as a consultant for the pharmaceutical industry and as an expert witness in a patent infringement case (2015).

Further to this, the authors also noticed two small errors, listed below:

1. The sentence beginning ‘Moreover, smoking prevalence remains disproportionately…’ should instead read as the following:

“Moreover, smoking prevalence remains disproportionately high in underrepresented (e.g. vulnerable/marginalised) groups (estimates of above 80% in those dependent on illicit substances) [11], and the effectiveness of e-cigarettes and other reduced risk nicotine-containing products have not been extensively researched in these groups.”

2. The sentence beginning ‘Why smokers with access …’ should instead read as the following:

“Why smokers with access to reduced risk products would still want to smoke and what factors encourage complete switching to reduced harm products are key areas requiring empirical investigation.”
